# Contributions to Our Journal by Societies

**Published:** 1841-07

**Authors:** 


### CONTRIBUTIONS TO OUR JOURNAL BY SOCIETIES.

It will be seen, that we are indebted to the *Medical Society of Tennessee* and to the *Louisville Disirict Medical Association*, for several original papers, published in this number. It is truly refreshing to find our physicians meeting for some other purpose than to enact laws against ungentlemanly conduct; or to listen to public *elementary* lectures on things which every student ought to know. The true ends of all Medical Associations appear to us to be the three following: First, that the physicians of the same country may become personally acquainted, and thus widen the sphere of their social enjoyments;—second, that they may plan and give an impulse to the execution of those works of benevolence and patriotism, which should originate with our profession:—that they may contribute to it the results of their observation, experience and experiments—to be discussed and published. Of these objects the last is unquestionably the most important; and all of them should be placed before blue-law legislation, and public *ad captandum vulgus* orations and addresses, composed of ideas, *uncommon* only in the fanatical concentration they may receive, or the new costume in which they may be arrayed. There is not in the "wide world" a nobler and richer field for original Medical and Physiological observation, than our beloved West and South present, and in every part of both, there are scientific physicians, who if they would diligently observe, and industriously communicate to each other, at stated times, the results of their observation, to be then and there scrutinized, would do more for the elevation of our professional character than could be accomplished in any other way. With these views before us, we respectfully urge upon all Societies and Associations now existing among us, or that may be, hereafter, formed, to turn their attention to this object, with renewed energy. We shall at all times be happy to give publicity to every paper which contains new facts or suggestions, which will at once make them extensively known, for our monthly reaches all the great divisions of the Mississippi Valley.

